# Microglial Activation and Inflammation as a Factor in the Pathogenesis of Progressive Supranuclear Palsy (PSP)

**DOI:** 10.3389/fnins.2020.00893

**Published:** 2020-09-02

**Authors:** Piotr Alster, Natalia Madetko, Dariusz Koziorowski, Andrzej Friedman

**Affiliations:** ^1^Department of Neurology, Medical University of Warsaw, Warsaw, Poland; ^2^Department and Clinic of Neurology, Wrocław Medical University, Wrocław, Poland

**Keywords:** PSP, microglia, neuroinflammation, neurodegeneration, progressive supranucelar palsy

## Abstract

Progressive supranuclear palsy (PSP) is a neurodegenerative disease based on four-repeat tauopathy pathology. Currently, this entity is not fully recognized in the context of pathogenesis or clinical examination. This review evaluates the association between neuroinflammation and microglial activation with the induction of pathological cascades that result in tauopathy pathology and the clinical manifestation of PSP. Multidimensional analysis was performed by evaluating genetic, biochemical, and neuroimaging biomarkers to determine whether neurodegeneration as an effect of neuroinflammation or neuroinflammation is a consequence of neurodegeneration in PSP. To the best of our knowledge, this review is the first to investigate PSP in this context.

## Introduction

Progressive supranuclear palsy (PSP) is among the most common types of atypical parkinsonism. In the contemporary literature, PSP has two meanings. The first is associated with tauopathy pathology and tufted astrocytes (Fernández-Botrán et al., 2011). Tufted astrocytes are characterized by the layout of tau, which are thin, long, and branching. This arrangement is observed from the cytoplasm to the proximal processes of astrocytes (Yoshida, 2014). In PSP, the neurofibrillary tangles are based on four repeat aggregates accompanied by glia in the basal ganglia and brain stem (Litvan, 2003). Recent studies have indicated that astroglial and oligodendroglial tau accumulation may be a key element in the pathogenesis of PSP (Höglinger et al., 2017). Researchers have found that these factors change neuronal tau pathology in the striatum, cortical regions, globus pallidus, and cerebellar white matter.

The second meaning of PSP is associated with PSP syndrome (PSPS), with reference to several correlated symptoms based on various pathologies, including PSP, corticobasal degeneration (CBD), and frontotemporal dementia (FTD). Oculomotor dysfunction (O), postural dysfunction (P), cognitive and language impairments (C), and akinesia (A) are among the major features of PSPS (Caso et al., 2018). Depending on the correlation between symptoms, several subtypes of PSPS have been characterized. The two most common variants are PSP-Richardson–Steele–Olszewski syndrome (affecting approximately 60% of cases) and PSP–parkinsonism predominant (affecting approximately 30% of cases) (McGeer et al., 1988). According to recent diagnostic criteria, the only definite diagnosis of PSP is based on autopsy, as the pathological pillars of this disease have only been partly elucidated. Furthermore, the correlation between PSP pathogenesis and microglial activation remains unclear. Moreover, microglial activation, inflammatory processes, and the microglial context in neuroimaging and biochemical examination are not currently included in the PSP guidelines (Caso et al., 2018).

The role of neuroinflammation in neurodegeneration was primarily investigated in research conducted by McGeer et al. (1988) in the late 1980s. This groundbreaking work highlights the significance of HLA-DR–positive microglia activation as an indicator of dopamine transporter and Parkinson-type pathology in the elderly. The theory proposed by McGeer et al. showed the association between microglial activation and neurodegeneration. The study found that a not fully recognized factor chronically impacted the tissue and activated microglial activation. An advancing pathophysiology was associated with the microglia, which remained activated after the destruction of the damaged elements of the dopaminergic system. The sustained activation of microglia is associated with the hypothesis of a neurodegeneration mechanism. This hypothesis is consistent with mechanisms observed in neurodegeneration triggered by exogenic factors, such as chronic traumatic encephalopathy (CTE), where the accumulation of pathological tau is gained due to the primary stimulation of head trauma. Within the course of CTE, the propelling mechanism of microglial activation results in progressive neurodegeneration. However, exogenic factors may also be associated with pathogens. The search for a link between neuroinflammation and parkinsonian syndrome was previously studied in the context of Spanish flu and its secondary disorders, such as encephalitis lethargica and postencephalitic parkinsonism (Papa et al., 2020). However, no proven correlation was found. The viral stimulation of microglial activation in neurodegeneration has recently gained attention as a key issue in the context of SARS-CoV-2 and COVID-19. Partly due to the lack of the possibility for long-term observations, recent studies have not indicated whether any correlation exists between COVID-19 and morbidity in parkinsonian syndromes (Papa et al., 2020). On the other hand, a study highlighting the impact of coronavirus on the central nervous system demonstrated the rapid spread of the virus in structures connected with the olfactory bulb, which included the basal ganglia, among which the ventral pallidum and lateral preoptic regions were mentioned. These regions were affected by an increased neuronal death (Netland et al., 2008). This effect of coronavirus infection indicates the potential for long-term consequences of COVID-19 (Lechien et al., 2020). Until now, no studies have focused on PSP despite the fact that these observations indicate the necessity of extended research concerning future diagnoses of PSP among patients primarily infected with SARS-CoV-2.

Research on neurodegeneration in Parkinson disease (PD) has found a correlation between neurodegeneration and abnormalities in the homeostasis of iron (Netland et al., 2008; Friedman and Galazka-Friedman, 2012; Lechien et al., 2020). The correlation of aberrations in iron, ferritin, oxidative stress, and Fenton reactions has been widely discussed in the context of PD (Friedman and Galazka-Friedman, 2012). In this context, the levels of anti-inflammatory cytokines, inflammatory cytokines, and NT-proCNP have shown significant abnormalities (Koziorowski et al., 2012). In PSP, abnormalities related to higher levels of total iron were observed within the substantia nigra (SN) and globus pallidus (Galazka-Friedman et al., 2012). These observations lead to the identification of regions of interest that were potentially more susceptible to neuroinflammatory factors. Moreover, another study analyzing angiogenesis in PD and PSP indicated the SN as a region of higher microglia number and microglial activation in both diseases (Desai Bradaric et al., 2012). This raises the question of determining which of the components is the cause and which is the effect. Thus, research on the role of iron concentration may be significant for the extended analysis of PD and parkinsonism-predominant PSP. The entities that show significant overlaps, especially in the primary years, are based on different mechanisms. The neuroinflammatory basis of neurodegenerative diseases may be associated with the iron abnormalities observed in the pathogenesis of PD and PSP. Recently, studies evaluating blood and imaging markers in parkinsonism have found significant differences in the differentiation of PD and APS. One study evaluated neurofilament light chain (NFL), a marker of neuroaxonal injury indicating neuroinflammation and planimetric measurement, and found that although the pontine-to-midbrain-diameter ratio differentiates PSP with PD and multiple system atrophy with predominant parkinsonism (MSA-P), the singular assessment of NLR showed significant differences only at the boundaries of PD and APS (Mangesius et al., 2020). This shows that the marker of neuroinflammation does not provide sufficient data for the most critical discrimination. The combined assessment was interpreted as class III evidence. The aim of this review is to discuss these features and analyze contemporary hypotheses concerning the pathophysiology.

## Link Between Neuroinflammation and Neurodegeneration

Microglia, an immune defense element in the central nervous system, represents 10–12% of the total brain’s cell population (del Rio-Hortega, 1932; Saeed et al., 2017). Unlike the majority of histological elements of the central nervous system, microglia do not evolve from the myeloid lineage of the neuroectoderm. The activation of microglia may be a result of various factors, including singular and multiple. For example, exogenous lipopolysaccharide, neural injury, and damage are among the factors initiating this activation. Activated microglia release various inflammatory factors, including tumor necrosis factor α (TNF-α), nitric oxide (NO), and reactive oxygen species (Lull and Block, 2010).

The inflammatory process resulting from microglia activation is part of the pathogenesis of PD and other parkinsonian syndromes (Yoshida, 2014). Activated microglia produce NO, which releases iron from ferritin (McGeer and McGeer, 2004). Another low molecule that can release iron from ferritin is ^⋅^O_2_^–^ (Reif and Simmons, 1990). The concentration of ^⋅^O_2_ increases after the blocking of mitochondrial complex I by toxic compounds, such as MPTP or mutated *PINK* protein (Reif and Simmons, 1990; Yoshida et al., 1995). Recent data have shown that the activation of microglia in neurodegenerative diseases and schizophrenia may be related to human endogenous retroviruses (HERVs) (Gruchot et al., 2019). Microglial cells are currently viewed as a relevant source of HERV proteins in the diseased brain. In this regard, it is worth mentioning that NO production and cellular migration were found to be affected in response to the stimulation of rat microglia with a recombinant HERV protein (Xiao et al., 2017). Pathological processes that use the dopaminergic system have diverse clinical manifestations. Psychotic disorders with positive or negative symptoms, including behavioral changes and altered cognitive abilities with speech disorganization may be associated with parkinsonian syndrome related to the degeneration of the dopaminergic system. Microglial activation has been correlated with the expression of leukocyte antigens, the upregulation of which is associated with neurodegenerative diseases (McGeer et al., 1993).

The activation of microglia in neurodegenerative diseases is associated with the release of various cytokines and chemokines. For example, the pathogenesis of PD is related to an increase in CCL2 (Grozdanov et al., 2014). This chemokine, which stimulates the evolution of myeloid cells from the bone marrow, is interpreted as a concomitant feature of an increase in circulating monocytes ratio (Fujimura et al., 2015). The activation of the triggering receptor of myeloid cells (TREM2) in PSP is associated with microglial activation and subsequent neurodegeneration (Leyns et al., 2017). On the other hand, the cytokines secreted by activated microglia, including TNF-α, interleukin 1β (IL-1β), and IL-6, are associated with neurodegeneration predominantly in atypical parkinsonism disorders, such as PSP and MSA (van Olst et al., 2020).

Studies on neurodegenerative diseases have yet to fully elucidate the role of microglial activation. The growth of cytokines or increase in CCL2 concentration is one of the features that may explain the role of microglia and microglial derivative factors. However, the associations between microglial activation and related features are more likely to be consequences of the pathomechanisms rather than the causes. Furthermore, the early features of microglia activation are not interpreted as defining targets of neuroprotective therapy. This suggests that the mechanism of microglial activation in neurodegenerative diseases is more complex than it appears.

## Tauopathy as a Cause of Neuroinflammation

The association between tauopathy and neuroinflammation has not yet been fully explored. Various studies have hypothesized that tauopathy may be the primary factor in neuroinflammation. In one study, tauopathy was investigated using mice models to verify the stage of initiation of tauopathy and to assess the related microglia activation. The study examined *Thy1-hTau.P301S (PS)* mice expressing human tau with a *P301S* mutation in the neurons and revealed the dystrophic complexity of cortical microglia following phosphorylated tau accumulation (Hickman et al., 2018). However, the impact of microglial activation on tau was not obvious and may result in the neutralization, disintegration, or deletion of tau and increase in tau phosphorylation (Lee et al., 2010; Asai et al., 2015; Bolós et al., 2016; Montagna et al., 2018). The majority of studies have shown a general association between microglial activation and tau pathology.

Research on different tauopathies has shown that various diseases arising on this pathology may be interpreted as a consequence of exogenic factors, inducing neuroinflammation or head trauma. Hypotheses of potential overlaps in the pathogenesis of PSP and other entities provide clues into the evolution of PSP.

Among these syndromes, IgLON-5–associated encephalitis has been linked to IgG1 class and inflammatory processes (Cagnin et al., 2017; Erro et al., 2019). Although the accumulation of phosphorylated tau varies between tauopathies, its partial resemblance and association with microglial activation provide insights into the role of microglial activation and inflammatory processes in PSP. IgLON-5–associated encephalitis may be related to the induction of microglial activation by perivascular CD8 T cells (Gaig et al., 2017). Interestingly, one of four patients described in a study examining anti–IgLON-5 manifested PSP-like syndromes (Mckee et al., 2018).

Another example of tauopathy encephalopathy is associated with CTE. Neurodegeneration with active microglia and inflammatory processes is associated with higher levels of cytokines in the brain tissue and cerebrospinal fluid (CSF) compared with primary tauopathies (Ling et al., 2015; Aldag et al., 2017; Makinde et al., 2017). This pathology is associated with neurofibrillary tangles. Clinical CTE is only partly associated with CTE pathology, as comorbid pathologies among patients with CTE are widely described. In one study, 37% of the patients did not have pure CTE pathology. Among comorbidities in the pathological analysis, the authors mentioned PSP, frontotemporal lobar degeneration, Pick disease and Lewy body disease (Cherry et al., 2016). Repeated traumatic brain injury results in an increase in the levels of IL-1, which is produced by activated microglia, resulting in increased tau phosphorylation. In a post-mortem study of 66 cases of CTE and 16 controls, the dorsolateral frontal cortex showed higher levels of CD68 immunoreactive microglia (Demock and Kornguth, 2019). Thus, CD68 was interpreted as being a potential therapeutic target for CTE. In another study, the release of interferon gamma from microglia was found to be a potential factor stimulating the higher expression of HLA class I proteins by neural cells. The redistribution of tau protein has been interpreted as a consequence of this process (Vogels et al., 2019).

The association between Alzheimer disease (AD) and microglial activation has also been highlighted. The correlation between spatial–temporal β-amyloid plaques and microglial activation has been evidenced in both patients and experimental models (Dani et al., 2018). Microglial activation and tau distribution have also been associated in AD (Taipa et al., 2018). The activation of microglia is also thought to be related to the pathogenesis of late-onset AD (LOAD) (Efthymiou and Goate, 2017; Bright et al., 2019). One study found increased microglial scores in the CA1 hippocampal subfield and entorhinal and temporal cortices, as well as a higher astroglial response in the CA1, dentate gyrus, entorhinal, and temporal cortices (Efthymiou and Goate, 2017). Among the primary tauopathies, microglial activation is also thought to be involved in the progression of FTD (Bolós et al., 2016; Marras et al., 2018).

Related pathological diseases have been found to overlap to a certain extent with the pathogenesis of PSP. However, compared to AD, the mechanism of neurodegeneration in PSP is less known. For example, the mechanism of neurodegeneration induced by cytokines stimulated by microglial activation is commonly used to explain various tauopathies.

Although PSP is clinically distinct from CTE and anti–IgLON-5 encephalopathy, it is clinically and pathologically associated with other tauopathy syndromes. A closer analysis of the diseases showed potential overlaps in their pathomechanisms, such as in the exogenic factors that initiate a neuroinflammatory response, which is secondary to neurodegeneration. The presence of similar clinical manifestations in PSPS, CTE, and anti–IgLON-5 encephalopathy, as well as other tauopathy syndromes, highlights the importance of identifying exogenic factors that promote neurodegenerative processes. Additionally, an overlap in the clinical manifestations of PSPS and anti–IgLON-5 encephalopathy and in the pathology of PSP and CTE demonstrate the necessity of combined analysis for elucidating the corresponding pathomechanisms.

## Biochemical Inflammatory Markers in Neurodegeneration

Microglial activation is associated with inflammatory processes. Thus, non-steroidal anti-inflammatory drugs (NSAIDs) have been investigated as a potential therapeutic strategy for PSP (Im et al., 2015). However, no associations between the use of NSAIDs and inflammatory processes in PSP have been observed.

Inflammatory genes have also been investigated as potential risk factors of PSP (Inci et al., 2020). A previous study investigated whether associations existed between neurodegeneration and inflammatory processes in PSP by evaluating the neutrophil-to-lymphocyte ratio (NLR) (Starhof et al., 2018). A significant increase in NLR was observed in the PSP group compared to the PD group and healthy volunteers. These results were interpreted as a confirmation of peripheral inflammation in PSP. The role of inflammation in the pathogenesis of PSP was also investigated by evaluating the expression of proinflammatory cytokines in various regions of the brain (Fernández-Botrán et al., 2011). As a result, an increase in the expression of IL-1β transcripts in the SN was associated with PSP. Changes were also observed in the parietal cortex in AD compared with PSP. Another study evaluated a panel of cytokines (interferon γ, IL-10, IL-18, IL-1β, IL-4, IL-6, transforming growth factor β1, and TNF-α) in the CSF of patients with PSP, MSA, and PD and found a significant increase in microglial-derived cytokines in PSP and MSA compared to PD (Hall et al., 2018).

The expression of inflammatory markers was analyzed in the CSF of patients affected by atypical parkinsonism disorders (Rydbirk et al., 2019). In a study conducted by Hall et al. (2018), the levels of IL-6 in CSF were found to be correlated with Unified Parkinson’s Disease Rating Scale Part III in patients with PSP. On the other hand, no significant correlation was observed using the PSP rating scale. These results indicated a potential correlation between increased inflammatory markers in MSA and PDD, indicating a potential high significance in α-synucleinopathies. However, this study was limited by a low number of examined patients (14 patients), which may impact the significance of the statistical assessment.

In a study highlighting the role of IL-2 in the pathogenesis of PSP, 16 patients with PSP and 16 controls were assessed. Based on the examination of 21 cytokines and growth factors, increased levels of IL-2 were observed in the PSP group. Based on these findings, other potential factors related to neuroinflammatory pathways were investigated, and an increase in the mRNA expression of glycogen synthase kinase 3β (GSK3B) was observed. IL-2 and GSK3B are interpreted as regulators of T and natural killer cells, which were previously identified as playing a role in neurodegeneration in PD and AD (Ishizawa and Dickson, 2001). The activation of microglia was verified in a study evaluating 10 patients with PSP and 5 patients with CBD via immunochemical post-mortem analysis (Bonham et al., 2018). Differences were observed in the microglia of patients with these tauopathies and the controls. Physiological concentrations of microglia were observed in the perivascular regions. In PSP and CBD, the microglia were observed within the pyramidal and extrapyramidal motor systems. In PSP, microglia were also observed in the cerebellar output. Additionally, the shape of cells was amoeboid and in certain regions aggregated, in contrast to the ramified cells in the controls.

At present, a definite mechanism for the immunological induction of neurodegeneration in PSP is lacking due to the multidimensional concepts underlying the pathogenesis of this disease. Thus, the role of microglial activation in this process remains to be fully elucidated. Furthermore, the current research has yet to determine whether neurodegeneration is a direct consequence of the neuroinflammatory processes in PSP.

Regardless of the ambiguity of the role of neuroinflammation, the impact of neuroinflammation on neurodegeneration is evident. The inhibition of mechanisms underlying neuroinflammation via microglial activation has been associated with beneficial effects on the pace and scale of morphological deterioration. The data show that analyzing the role of neuroinflammation in neurodegeneration will allow for a closer examination of the pathomechanisms of atypical parkinsonism disorders, rather than differential diagnoses. The contemporary literature indicates that inhibiting the cascades of reactions at various levels, such as the gene expression and secretion of microglial derivative cytokines, including IL-6 or TNF-α, may be beneficial in delaying these pathomechanisms. Studies have shown that the pathomechanisms of atypical parkinsonism disorders have a tendency to overlap somewhat. Future studies will need to determine why often similar mechanisms result in entities based on different pathologies, as in MSA and PSP.

## The Genetic Context of Microglial Activation

Microglial activation in the pathogenesis of neurodegenerative diseases was assessed on the basis of the association between the *CXCR4* gene and chemokine receptors and the microglial genes *CXCL12, TLR2*, *RALB*, and *CCR5* (Conway et al., 2018). Increased *CXCR4* expression was observed in the cerebellum of patients with PSP and FTD. Among the microglial genes, increased *CXCL12* expression has been interpreted as the most discriminating factor of PSP. Evaluating *CXCR4* and the microglial activation marker *TMEM119* revealed a correlation of limited significance. *CXCR4* was found to be related to neurodegeneration in PSP and other entities, such as PD and FTD. Increased *CXCR4* expression in the pathogenesis of the disease significantly overlaps with immune response signaling and the course of neurodegeneration.

Another study showed that a microglial gene–enriched coexpression network related to *ABI3* and *PLCG2* was significantly increased in AD, but not in PSP (Sánchez-Ruiz de Gordoa et al., 2020). The microglia-related gene triggering receptor in myeloid cells (TREM2) has also been investigated in the context of PSP (Desai Bradaric et al., 2012). Higher levels of *TREM2* and its three *TREM2* transcripts were observed in the SN. *TREM2* mRNA levels were also positively correlated with hyperphosphorylated tau burden in the SN. The activation of microglia in the SN of patients affected by PSP was also observed in another study (Shi et al., 2019). The expression of *TREM2* was also investigated in a study evaluating the impact of TREM in developing AD in experimental models. In this study, the inhibition of microglial TREM2 signaling was associated with reduced inflammation (Leyns et al., 2017). This process was found to provide protection against neurodegeneration based on tauopathy. A protective role was also observed in the maintenance of volume in the entorhinal and piriform regions. This study examined mice expressing human mutated tau (*P301S*). The involvement of microglial activation in neurodegeneration was also investigated in a study highlighting microglia drive APOE-dependent neurodegeneration (Gerhard et al., 2006). *APOE*, which is widely known as a risk factor of AD, was found to be a critical factor in stimulating neurodegeneration in the process of impacting microglial activation. Microglial activation, unlike microglial proliferation, was linked with neurodegeneration. Herein, deficiencies in *APOE* were found to be crucial in its protective role.

The genetic context of neuroinflammation in neurodegeneration is characterized by correlations, which may help in understanding the processes leading to tauopathy in the context of triggering receptors in myeloid cells. Several factors, including *CXCL12*, *TLR2*, *RALB*, and *CCR5*, have been associated with microglial activation, a proven feature of neurodegeneration. The expression of factors such as CXCL12, a differentiation factor in PSP, presents a basis for further evaluations regarding the genetic basis of neuroinflammation in neurodegeneration rather than a breakthrough.

## Neuroimaging

Microglial imaging is based on the affinity between the radiotracer used in positron emission tomography (*^11^C-PK11195*) and mitochondrial translocator protein (TPSO) (Saeed et al., 2017). The upregulation of TPSO is associated with increased microglial activation, which has been observed in neurodegenerative processes.

Microglial activation has been associated with PSP in various contexts. In neuroimaging, this was investigated by assessing radiotracers in post–iron emission tomography. To this end, Gerhard et al. (2006) assessed the role of ^11^C-PK11195 in various parkinsonism disorders. In PSP, increased binding was observed in the basal ganglia, midbrain, frontal lobe, and cerebellum. In a study conducted by Passamonti et al. (2018), an increased accumulation was observed in the putamen, thalamus, and pallidum. The binding in the pallidum, midbrain, and pons was interpreted as a correlation with the severity of the disease (Passamonti et al., 2018). The neuroimaging of inflammation has been used to determine whether brain inflammation is correlated with cognitive impairments in memory disorders using the microglial activation radiotracer ^11^C-PK11195. Additionally, the concomitant use of C-labeled Pittsburgh Compound B ([^11^C]PiB) and ^18^F-labeled AV-1451 will allow researchers to determine whether β-amyloid and tau accumulation are associated with microglial activation (Bevan-Jones et al., 2017).

The lack of a definitive mechanism for neurodegeneration or its correlation with microglial activation has limited the significance of the study of PSP in clinical practice. However, the correlation between the *in vivo* examination of tau and microglial activation demonstrates the importance of further analyses on this issue. An association between psychopathy and microglial activation has been demonstrated by examining patients using microglial activation (^11^C-PBR28) PET and tau (^18^F-AV1451) PET radiotracers. To this end, the presence of β-amyloid was assessed *in vivo* using ^18^F-flutemetamol, confirming that microglial activation is correlated with both β-amyloid and tau distribution. Microglial activation has also been associated with synapse loss and the exacerbation of tau pathology (Hansen et al., 2018).

Currently, single microglial activation PET seems to be an insufficient tool in neuroimaging. By contrast, parallel examinations using different radiotracers, such as ^18^F-AV1451 and ^11^C-PK11195, may provide relevant methodologies for evaluating advanced clinical syndromes. However, they have not yet managed to elucidate the pathomechanisms of the entities.

## Conclusion

The pathogenesis of PSP has yet to be fully elucidated because of issues concerning possible correlations between inflammation and microglial activation. Recent studies have shown that the pathomechanism of PSP differs significantly from that of PD. However, whether the four repeated tauopathies arise from the effects of the same inflammatory processes or whether these processes overlap fully should be investigated in future studies. This review shows that microglial activation in PSP is associated with the pathogenic deposition of tau. Despite multiple hypotheses, the mechanism of neurodegeneration in this disease has not yet been clarified. The mechanisms underlying PSP have been found to overlap with PD, namely, the activation of microglia in the SN. However, other elements, such as the profiles of inflammatory factors in the CSF in PD and PSP, suggest that the mechanisms underlying microglial activation and inflammatory processes in both diseases differ significantly. Further research will be needed to verify our findings and fully elucidate these mechanisms.

## Author Contributions

PA: study design, data analysis, review of the literature, and final acceptance. NM and DK: data analysis, review of the literature, and final acceptance. AF: review of the literature and final acceptance. All authors contributed to the article and approved the submitted version.

## Conflict of Interest

The authors declare that the research was conducted in the absence of any commercial or financial relationships that could be construed as a potential conflict of interest.
